# Gender matters, especially if you are a Polish teenager being examined by a doctor or a doctor examining a teenager

**DOI:** 10.1007/s00431-024-05778-y

**Published:** 2024-10-19

**Authors:** Michał Henzler, Ania Stella Henzler, Jan Jacewicz, Aneta Nitsch-Osuch, Ernest Kuchar, Anika Rybicka-Kramarz, Bartłomiej Kucharski, Magdalena Bogdan

**Affiliations:** 1 Department of Social Medicine and Public Health, Medical University of Warsaw, ul. Oczki 3, 02-007 Warsaw, Poland; 2 Warsaw, Poland; 3 Department of Pediatrics, Clinical Assessment Unit, Medical University of Warsaw, Warsaw, Poland

**Keywords:** Adolescent-friendly health services, Adolescent medicine, Gender inequality, Genital examination, Well-care visits

## Abstract

To analyse how the specific gender configurations of the adolescent patient and the doctor affect the performance of intimate areas examinations during adolescent well-care visits and the emotions that accompany these examinations, the use of comfort measures, and the subsequent willingness of adolescents to visit the doctor in the future. An anonymous questionnaire was completed by adolescents (*n* = 1072) and their parents (*n* = 685) recruited from 80 randomly selected secondary schools in Poland. Genital examination and puberty assessment were performed more often in boys than in girls (OR = 14.1, *p* < .0001 and OR = 5.5, *p* < .0001, respectively). Female doctors performed intimate examinations more frequently than male doctors (OR = 2.2, *p* = .0059). Male doctors were more likely to ask for consent than female doctors, 66.7% vs. 40.2% (*p* = .0556), and use the screen more often than female doctors, 46.7% vs. 21.7% (*p* = .0393). Intimate examinations significantly discouraged adolescents from revisiting the doctor, especially when performed by a physician of the opposite sex. The majority of Polish adolescents and their parents believe that routine prophylactic genital region examinations are illegal, especially if girls’ genitals are to be examined by male physicians.

*Conclusion*: The implementation of preventive genital region examinations in adolescents depends largely on non-medical factors—the gender of the patient, the physician, and the mutual configuration of these genders. The level of public non-acceptance of these procedures should not be underestimated by experts setting standards of well-care for adolescents.

**Table:** 

**What is known:** *• Although the assessment of sexual maturity is an integral part of the preventive examination of adolescents (both girls and boys) these examinations are often overlooked.* **What is new:** *• The gender of the physician and the congruence of his/her gender with the gender of the adolescent patient influence whether a puberty assessment will be performed, as well as the social acceptance of such procedures.* *• Examinations of the intimate area are a significant factor discouraging both adolescent girls and adolescent boys from seeing the doctor again.*

**Supplementary Information:**

The online version contains supplementary material available at 10.1007/s00431-024-05778-y.

## Introduction

Several studies show that the gender of the physician, as well as the congruence (or incongruence) of that gender with the gender of the patient, affects the quality of medical services provided [1–4]. In previous studies, we have shown that a compatible configuration of physician and patient gender, for example, promotes more topics covered during an adolescent well-care visit, whereas a female patient’s gender reduces the likelihood that examinations requiring increased body exposure will be performed [5]. These findings inspired us to investigate the influence of the gender configuration of the physician and the adolescent patient on the implementation of preventive examinations of the genital region, which, in line with standards in Poland and many other countries, are an integral part of an adolescent well-care visit.

An additional goal of this study was to provide insights into how genital region examinations affect visit evaluations and adolescents’ willingness to visit a doctor in the future, as well as whether the procedure is perceived by Polish society as legally acceptable.

The issue of the emotional and social context of prophylactic examinations of the genital area in adolescents (including assessment of puberty) is hardly represented in the medical literature [6, 7], despite the fact that in many countries, these procedures are controversial in both society and professional circles. In France, for example, routine prophylactic examination of girls’ intimate areas is considered by some feminist circles to be a manifestation of a kind of obstetric violence [8], and in Sweden, there is debate as to whether routine examination of the genital area is ethically acceptable even as part of measures to prevent genital mutilation in girls [9].

## Materials and methods

An anonymous survey was conducted among students aged 14–17 years from 80 randomly selected Polish secondary schools in 13 voivodeships (equivalent to provinces)—as well as among parents of students from these schools.

Randomly selected schools involved in the survey received online links to two questionnaires—one for students and one for their parents. The schools forwarded both links only to the parents. The parent could complete the questionnaire themselves and/or forward the relevant link to the questionnaire to her/his teenager. At the same time, he/she had the opportunity to review the content of the questionnaire addressed to the teenagers and give informed consent for his/her child to participate in the survey.

Thanks to this procedure, consent to participate in the study was obtained from not only the participants but, in the case of underage participants, also their parents. The study and the procedure for obtaining dual consent were approved by the Bioethics Committee of the Medical University of Warsaw. The questionnaire was returned by 1796 respondents. Thirty-three questionnaires that lacked information on the gender of the adolescent and six questionnaires where the age of the adolescent was not between 14 and 17 years were excluded from the analysis. In total, the responses from 1072 adolescents and 685 parents were analysed.

For the demographic profile of sample groups, see Supporting information.

### Questionnaires

The questionnaire addressed to teenagers included 77 close-ended questions mainly about the course of the last well-care visit attended by the adolescent surveyed and a number of questions on gender-sensitive aspects, including, for example, preferences regarding the gender of the physician performing examinations, physicians’ use of procedures that protect the adolescent’s intimacy (e.g. use of a screen), and adolescents’ and their parents’ knowledge of the legality of genital examinations.

The questionnaire addressed to parents contained 38 closed questions, including those regarding parents’ opinions on the legality of preventive examinations of the genital areas in adolescents, depending on the gender of the physician and the gender of the examined adolescent.

Both questionnaires also contained a number of questions not directly related to the subject of this paper, but raising other aspects of the preventive visit. Our previous experience showed that questionnaires containing only questions about sex, gender, and examination of genital areas in the rather conservative Polish society may arouse controversy and reduce the percentage of schools and parents who will consent to the participation of teenagers in the survey.

For detailed information about analysed gender-sensitive aspects and the questionnaire, see Supporting information.

### Statistical analyses

Data were analysed using Statistica 13.3 (TIBCO Software). As none of the data sets met the criteria for normal distribution (Shapiro–Wilk test), non-parametric tests were used to process the data, including Pearson’s chi-squared tests, Fisher’s exact tests, Mann–Whitney *U* tests, and Kruskal–Wallis *H* tests. Values were considered significant where *p* ≤ 0.05.

## Results

### Implementation of standards

Of the adolescents surveyed, 626 (58.4%) had a well-care visit in the past 18 months. There were no differences by gender (*p* = 0.9573).

#### Effect of patient gender

Examination of intimate regions was performed in 107 (17.1%) adolescents. Of these, 107 were assessed for pubertal stage according to the Tanner scale, and 41 underwent examination of external reproductive organs that included testicular palpation or manual exposure of the vaginal vestibule. Pubertal stage scale assessment was performed more frequently in boys than in girls (OR = 5.5; 95% CI 3.4, 8.4), during 32.1% vs. 8.2% of visits, respectively (${\chi }^{2}$[1, *n* = 626] = 59.00, *p* < 0.0001). Examination of external genitalia was also performed more frequently in boys than in girls (OR = 14.1; 95% CI 5.4, 36.4), during 15.4% vs. 1.3% of visits, respectively (${\chi }^{2}$[1, *n* = 626] = 47.66, *p* < 0.0001). Patient gender was not found to influence the frequency of abdominal (62.6%) or spine (87.2%) examinations. Girls were auscultated with a stethoscope slightly more often than boys, during 91.6% vs. 86.8% of visits, respectively (${\chi }^{2}$[1, *n* = 626] = 3.72, *p* = 0.0537).

#### Influence of physician gender

A visit to a female doctor was associated with a higher likelihood of examining intimate areas than a visit to a male doctor (OR = 2.2; 95% CI 1.3, 4.0). Pubertal stage assessment was performed during 19.5% of visits to female doctors and during 9.8% of visits to male doctors (${\chi }^{2}$[1, *n* = 626] = 7.59, *p* = 0.0059), and genital examinations were performed during 7.6% of female physician visits and during 3.3% of male physician visits (${\chi }^{2}$[1, *n* = 626] = 3.56, *p* = 0.0591) (see Table 1, part A).

**Table 1:** Implementation of selected elements of the well-care visit, as well as the assessment of its parameters and consequences, by adolescents subjected and not subjected to examinations requiring exposure of the genitals

	A girl + a female doctor	A boy + a female doctor	A boy + a male doctor	A girl + a male doctor	Statistical significance
A. Implementation of “intimate” examinations among all participants of well-care visits (*N* = 626) (percentage of visits where the examination was conducted)					
Number of respondents in sample by specific gender configuration	*n* = 307	*n* = 166	*n* = 68	*n* = 85	
Pubertal stage assessment	10.1%	36.8%	20.6%	1.2%	${\chi }^{2}$(3, *n* = 626) = 71.63, *p* < 0.0001
Examination of genitalia	1.6%	18.7%	7.4%	0%	${\chi }^{2}$(3, *n* = 626) = 58.05, *p* < 0.0001
B. Respect for intimacy—“intimately” examined adolescents (*N* = 107) (percentage of visits where the procedure was used)					
Number of respondents in sample by specific gender configuration	*n* = 31	*n* = 61	*n* = 14	*n* = 1	
Patient was asked for consent before the intimate examination	61.3%	29.5%	64.3%	Was asked	${\chi }^{2}$(6, *n* = 106) = 13.74, *p* = 0.0327
Patient was shielded by a screen for examination	35.5%	14.8%	42.9%	Was shielded	${\chi }^{2}$(6, *n* = 106) = 18.84, *p* = 0.0266
C. Emotions and consequences—“intimately” examined adolescents (*N* = 107) (mean value on 1–5 Likert scale)					
Number of respondents in sample by specific gender configuration	*n* = 31	*n* = 61	*n* = 14	*n* = 1	
Assessment of physician’s respect for patient’s intimacy	3.13 (SD 1.1)	2.75 (SD 1.2)	3.4 (SD 0.9)	Score “1”	*H* (3, *n* = 106) = 5.2828, *p* = 0.0713
Impact on adolescent’s motivation for the next visit	2.6 (SD 1)	2.4 (SD 0.8)	3.1 (SD 0.5)	Score “2”	*H* (3, *n* = 106) = 6.5793, *p* = 0.0373
D. Emotions and consequences—“non-intimately” examined adolescents (*N* = 519) (mean value on 1–5 Likert scale)					
Assessment of physician’s respect for patient’s intimacy	3.8 (SD 1) — no statistically significant differences according to the gender configuration				*H* (3, *n* = 519) = 9.9961, *p* = 0.3922
Impact on adolescent’s motivation for the next visit	2.95 (SD 0.7) — no statistically significant differences according to the gender configuration				*H* (3, *n* = 519) = 2.10495, *p* = 0.5509

Intimate examination = pubertal stage assessment (according to the Tanner’s scale) and/or full external genitals examination

Physician gender was not found to influence the frequency of abdominal or spine examinations. Female physicians auscultated the chest slightly more frequently than male physicians—91.3% vs. 85%, respectively, (${\chi }^{2}$[1, *n* = 626] = 5.10, *p* = 0.0239).

### Respect for intimacy

Of the 107 adolescents who underwent genital region examinations, 47 (43.9%) were asked for consent beforehand. The others were examined “by surprise” or under a sense of “coercion”. Consent was significantly more often asked from girls than boys—66.5% vs. 36%, respectively (${\chi }^{2}$[1, *n* = 107] = 6.39, *p* = 0.0114). Male doctors were more likely to ask for consent than female doctors—66.7% vs. 40.2%, respectively (${\chi }^{2}$[1, *n* = 107] = 3.66, *p* = 0.0556).

Doctors shielded 37.5% of the girls and 20% of the boys with screens during intimate examinations (${\upchi }^{2}$[1, *n* = 107] = 3.64, *p* = 0.0564). Male doctors used the screen significantly more often than female doctors—46.7% vs. 21.7%, respectively (${\chi }^{2}$[1, *n* = 107] = 4.25, *p* = 0.0393) (see Table 1, part B).

### Emotions and their consequences

#### The effects of examining the genital region

Adolescents who underwent intimate region examinations were more likely to rate their most recent visit “very poorly” or “poorly” in the “respect for intimacy” category (1 or 2 points on a 5-point scale) than non-examined adolescents (OR = 9.21; 95% CI 5.24, 16.21). Adolescents who received an intimate examination at their last well-care visit were more likely to say they felt “very discouraged” or “discouraged” from attending another visit (OR = 4.83; 95% CI 3.01, 7.76).

#### The effect of gender configuration

The proportion of negative or very negative ratings in the “respect for intimacy” category was higher among adolescents examined by a doctor of the opposite gender than among adolescents examined by a doctor of the same gender (OR = 3.71; 95% CI 1.43, 9.64). Adolescents who had their genitals examined by a doctor of the opposite gender were more likely to report being “discouraged” or “very discouraged” from attending the next visit than adolescents who had their genitals examined by a doctor of the same gender (OR = 2.92; 95% CI 1.29, 6.62) (see Table 1, parts C and D).

### Social convictions

A sizeable proportion of respondents mistakenly believed that the Polish law prohibits preventive examinations of adolescents’ external genitalia, in particular, by doctors of a gender incompatible with the adolescent’s sex.

All groups of respondents—girls, boys, and parents—were convinced that the law provides greater “protection” against genital examination by a doctor of a different sex for girls than for boys.

All groups of respondents were convinced that male doctors have less right to examine girls’ genitals than female doctors to examine boys’ genitals. The mentioned differences are statistically significant (*p* < 0.05).

**Fig. 1:**
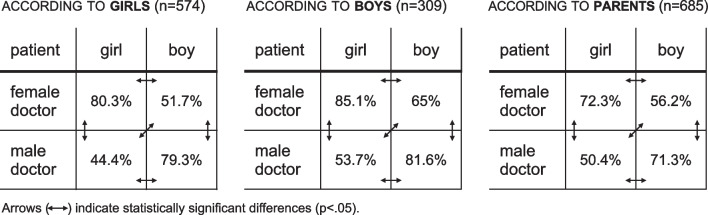
Beliefs on the permissibility (under the law) of prophylactic genital examination in adolescents depending on the gender of the patient and the gender of the physician. The figures in the tables outline the percentage of respondents who believe that, in a given gender configuration, preventive genital examination is permissible under the law

Almost 30% of parents believed that prophylactic examination of the external genitalia in adolescents is prohibited by law (see Fig. 1).

### Physician gender preference

The importance attached by adolescents to the gender of the physician depends on gender and the nature of the examination. Of the four procedures analysed (weight measurement, chest auscultation, spine examination, genital examination), the least gender-sensitive appears to be the spine examination—65.7% of girls and 72.2% of boys declared no preference for the gender of the doctor (${\chi }^{2}$[1, *n* = 883] = 3.88, *p* = 0.0488). The most gender-sensitive was the genital examination—only 9.1% of girls and 27.2% of boys declared no preference for the doctor’s gender (${\chi }^{2}$[1, *n* = 883) = 50.65, *p* < 0.0001).

#### Gender of the adolescent

**Fig. 2:**
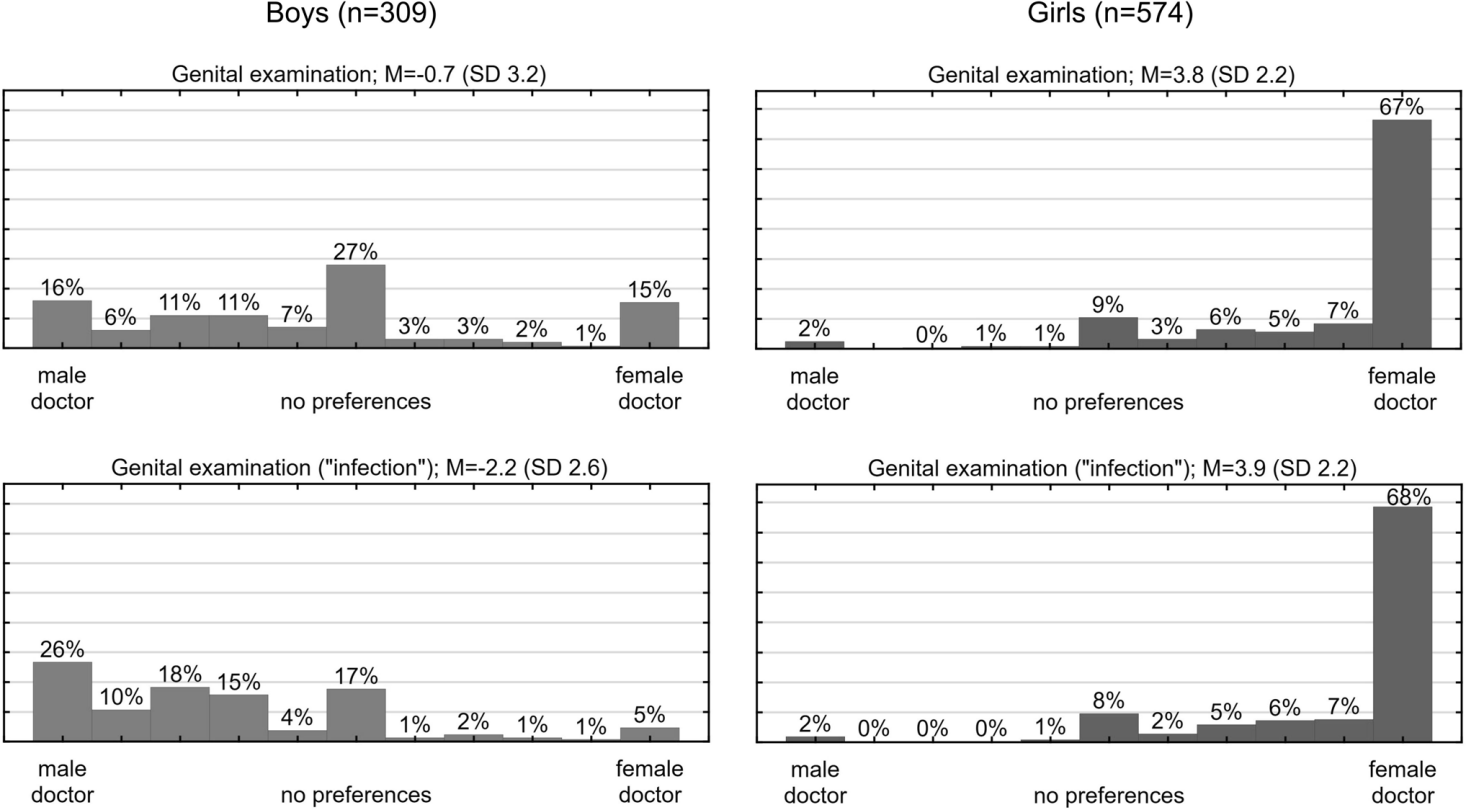
Distribution of adolescent preferences on the gender of the doctor conducting examinations (healthy and in the case of infection). The respondents indicated the preferred gender and the strength of this preference on a scale from − 5 to 5 (with zero indicating no preference, scores less than zero indicating a male preference, and scores greater than zero indicating a female preference)

87.1% of girls and 49.5% of boys preferred to have their genitals examined by a doctor of the same sex (${\chi }^{2}$[1, *n* = 883] = 147.37, *p* < 0.0001). In the hypothetical situation of an abnormality involving the genitals, this percentage of boys increased to 72.8% (${\chi }^{2}$[1, *n* = 309] = 35.31, *p* < 0.0001), but no statistically significant change was observed for girls in an analogous situation (see Fig. 2).

For more information about adolescents’ preferences, see Supporting information.

#### Age of the adolescent

**Fig. 3:**
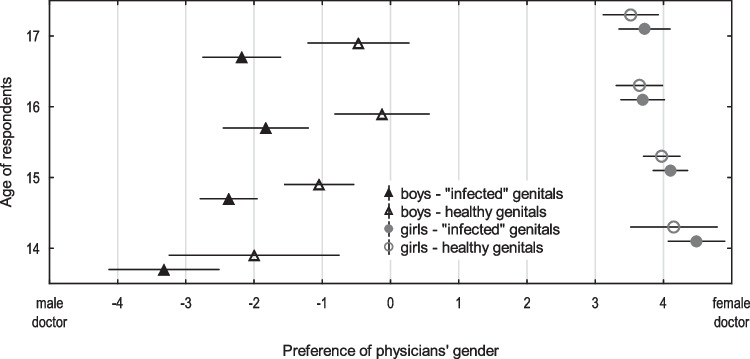
Influence of age and sex of adolescents on their preferences for the gender of the doctor conducting examinations of the genitals (healthy and in the case of infection). The graph shows the mean value (triangles and circles) of the preference assessment on a scale from − 5 to 5 (with zero indicating no preference, scores less than zero indicating a male preference, and scores greater than zero indicating a female preference). Horizontal line = standard deviation

Among younger adolescents (14–15 years old), a higher percentage of respondents indicated that they preferred intimate examinations be performed by a doctor of the same sex than among older respondents (16–17 years old). This effect was particularly pronounced for boys—56.5% (younger) vs. 43.9% (older; ${\chi }^{2}$[1, *n* = 309] = 4.9, *p* = 0.0269), and in girls, it was not statistically significant (*p* = 0.1058) (see Fig. 3).

## Discussion

Although Polish standards require an assessment of pubertal stage and external genitalia examinations in all adolescents during well-care visits [10], examinations of the genital regions were performed many times less frequently than examinations not involving intimate areas [5].

It should be noted that omissions with regard to standard-required examinations of intimate regions in adolescents are also observed, e.g. in the US [11] and in Italy [12]. In France, many paediatricians admit that instead of a full clinical examination, they only carry out “un examen clinique discret”—for example, they discreetly (through underwear) estimate testicular volume in boys [13].

This situation may be a result of both doctors’ lack of knowledge of the standards and/or a lack of subjective conviction about the benefits of these examinations. Insufficient skills in examining external genitalia in children [14, 15] and difficulties in overcoming one’s own embarrassment [16] cannot be excluded as an important barrier as well.

It is worth noting that the omissions of such examinations shape the public’s perception that such procedures are not a medically justifiable norm. It is therefore not surprising that a considerable proportion of Polish society believe that prophylactic genital region examinations in adolescents are an illegal activity.

### Implementation of standards

#### Gender of the patient and gender of the doctor

The current study confirmed that the implementation of preventive genital examinations depends largely on the gender of the patients, the gender of the practitioners, and the mutual configuration of these genders. Examinations of intimate areas were performed many times more frequently in boys. This inequality contradicts the Polish [10] and, for example, American (US) standards [17, 18] and may be the result of cultural conditions that traditionally guarantee special protection for the modesty of girls.

In the medical literature, the (theoretically) neutral act of genital examination of women and girls is given cultural significance. For example, the first pelvic examination is presented as a kind of rite of passage from childhood to adulthood and an emotionally charged moment [19–21]. In contrast, it is difficult to find publications that deal with the psychological or cultural dimensions of genital examinations in boys.

We also revealed that male physicians perform genital region examinations less frequently than female physicians. Similar observations have been made previously, for example, in the US [22]. In Poland, primary care paediatrics is a feminised specialty, and female paediatricians can be perceived as an “extension of motherhood” [23]. It cannot be ruled out—as has been described regarding to male nurse practitioners—that their professional actions are attributed by a part of society to a sexual context [24]. Fear of being suspected of non-medical motivations seems to be a likely reason for some male physicians to forgo genital region examination in adolescents. Additionally, male physicians during their studies and residency tend to have fewer opportunities to practically learn how to examine female genitals and are more often refused by female patients [25, 26].

### Respect for intimacy

A high percentage of adolescents surveyed underwent intimate examinations without being asked for consent (56.1% of those subjected to such examination), as well as in the presence of a parent, but without the use of a screen (74.8%). Such unacceptable practices are more common in boys.

It is worth noting that in many cultural circles, the very onset of puberty places the girl in certain gender-conditioned “adult” roles [27]. A pubescent girl may be perceived as a (little) woman, and the pubescent boy still as a (big) child. This phenomenon may be one of the reasons for less respect for boys’ need for intimacy; another may be the different manner in which gynaecology and urology are usually taught in medical schools [25, 26].

### Emotions and their consequences

We have shown that examinations of the intimate area—especially if performed by a doctor of the incompatible gender—are procedures that increase the likelihood that an adolescent will be discouraged from future visits to the doctor.

Although a comprehensive interpretation of adolescents’ preferences is beyond the scope of this publication, two of our findings should be noted:

1. Younger adolescents (14–15 years old) are more likely than older adolescents (16–17 years old) to prefer to have their genitals examined by a doctor of the same gender and declare that this preference is of greater importance to them.
2. For boys experiencing a possible abnormality in the functioning of the genitals, the compatibility of the genders is more important than usual.

### The need for reflection and action

In Poland, physicians are systematically boycotting genital region examinations in adolescents, and, at the same time, a significant part of the population considers these procedures illegal. In such a sensitive situation, it is necessary to take measures that protect both the interests of adolescents and the medical practitioners, who, by attempting to examine a teenager from head to toe, risk suspicion of non-professional motivations and loss of good reputation.

Firstly, it is essential to prove that the benefits of performing genital area screening in asymptomatic adolescents outweigh the losses, e.g. in terms of discouraging them from future contact with medical professionals. This is not so obvious. Some of the procedures, which until recently were seen as an essential part of well-care visits, are questionable in the light of current data, either because they have almost no impact on the long-term prognosis of patients (e.g. in the case of preventive testicular palpation to detect tumours) [28] or because they lead to overdiagnosis and exposure to potentially harmful medical tests—for example, X-rays when verification of suspected scoliosis is required [29].

It cannot be ruled out that screening of the genital area—even if fully medically justified—is unacceptable in certain societies and thus impossible to implement. Similarly, even in an accepting society, there are still individuals who will not consent to such screening. Therefore, current standards of well-care visits should be supplemented with clear instructions for practitioners on how to act in cases of lack of consent. It is worth noting that at least several alternative methods are being considered for assessing the progression of puberty based on indirect signs or based on self-assessment, such as the Pubertal Development Scale [30, 31].

It should be also emphasised that limitations on the part of medical doctors (e.g. lack of competence), incomplete acceptance on the part of the society, and respecting the right of adolescents to intimacy should not automatically lead to the elimination of genital examinations from the standards of preventive care. However, if these examinations are to remain a key element of an adolescent’s well-care visit, efforts are needed to change their image. These procedures must be perceived by adolescents, their parents, and the rest of society as a kind of offer by the medical practitioner to respond to the possible needs of the young patient.

It should be borne in mind that all adolescent genital examinations—also including those carried out by specialists (endocrinologists, surgeons, etc.)—are a special procedure with a high risk of violating the patient’s sense of dignity. As minors, adolescent does not have full legal authority to decide whether, by whom, or how he/she is examined. This incapacitation should oblige all healthcare providers who seek to provide adolescent-friendly healthcare to be particularly attentive to the patient’s sense of dignity and respect for his/her privacy, regardless of the patient’s gender, the practitioner’s gender, or the compatibility of them both.

## Supplementary Information

Below is the link to the electronic supplementary material.

Supplementary file1 (DOCX 391 KB)

## Data Availability

No datasets were generated or analysed during the current study.

## Abbreviations

CI: Confidence interval
FGM: Female genital mutilation
M: Mean
OR: Odds ratio
SD: Standard deviation
