# Supplementary material for: Gender matters, especially if you are a Polish teenager being examined by a doctor or a doctor examining a teenager
Source: Eur J Pediatr. 2024 Oct 19;183(12):5129–37. doi: 10.1007/s00431-024-05778-y (PMC11527909; doi:10.1007/s00431-024-05778-y)
Supplement: Supplementary file 1 — Supplementary file1 (DOCX 391 KB) [file 431_2024_5778_MOESM1_ESM.docx]

# Supplementary material

### Demographic profile of adolescents group in sample (n=1072).

| **variables** | | **n** | **%** |
| --- | --- | --- | --- |
| gender | girls | 672 | 62.7 |
|  | boys | 400 | 37.3 |
| age | 14 years old | 73 | 6.8 |
|  | 15 years old | 368 | 34.3 |
|  | 16 years old | 392 | 36.7 |
|  | 17 years old | 239 | 22.3 |
| place of residence | voivodship town | 123 | 11.5 |
|  | county town or town with county rights | 420 | 39.2 |
|  | town other | 107 | 10.0 |
|  | village | 422 | 39.4 |
| school type | vocational | 72 | 6.7 |
|  | technical school | 322 | 30.0 |
|  | high school | 678 | 63.2 |
| participation in well-child visit in last 18 mc | yes | 626 | 58.4 |
|  | no | 446 | 41.6 |

The sampling method was chosen to guarantee access to a representative group of respondents. Due to the fact that the authorities of the selected schools often refused to allow the research to be conducted, **the data obtained can be considered only very close to being representative of the population of underage Polish secondary school students**. A slight over-representation of girls and high school students is noticeable. In the opinion of the authors of the publication, **this fact does not affect the credibility of the data analyzed**, the more so as data for girls and boys are presented separately.

### Demographic profile of parents group in sample (n=685).

| **variables** | | **n** | **%** |
| --- | --- | --- | --- |
| adolescent’s gender | girl | 441 | 64.1 |
|  | boy | 244 | 35.6 |
| adolescent’s age | 14 years old | 57 | 8.3 |
|  | 15 years old | 273 | 39.9 |
|  | 16 years | 213 | 31.1 |
|  | 17 years | 142 | 20.7 |
| adolescent's place of residence | voivodship town | 90 | 13.1 |
|  | county town or town with county rights | 242 | 35.3 |
|  | town other | 62 | 9.1 |
|  | village | 291 | 42.5 |
| adolescent's type of school | vocational | 9 | 1.3 |
|  | technical school | 135 | 19.7 |
|  | high school | 540 | 78.8 |
| parent’s gender | mother | 639 | 93.3 |
|  | father | 46 | 6.7 |

The overrepresentation of mothers is a result of their typically and traditionally (in Poland) greater involvement in the education and health of children and adolescents. Similar to the adolescent population, there is an overrepresentation of parents of girls and high school students.


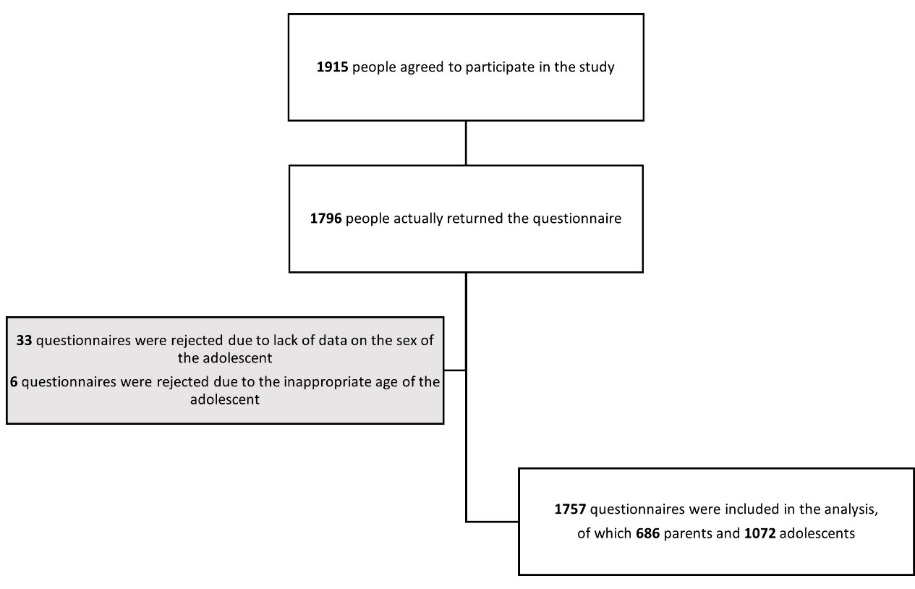


### gender-sensitive aspects

DIMENSION OF STANDARDS IMPLEMENTATION

The effect of patient gender, physician gender, and their compatibility or incompatibility on the performance of selected procedures required by the standards: (1) chest auscultation; (2) abdominal palpation, (3) postural examination, and (4) pubertal stage assessment and (5) general pediatric genital examination.

DIMENSION OF RESPECT FOR INTIMACY

The effects of patient gender, physician gender, and their configurations on (6) the frequency with which a physician obtains an adolescent's consent to conduct an "intimate examination"; and on (7) the frequency with which a screen is used during such an examination.

DIMENSION OF EMOTIONS AND THEIR CONSEQUENCES

The effect of being subjected to "intimate examinations" on (8) the subjective evaluation of respect for intimacy during a well-child visit, and to determine whether this effect depends on the patient's and physician’s gender, and their configuration.

The impact of "intimate examinations" on (9) adolescents' stated motivation to attend subsequent medical well-child visits, and to determine whether this impact depends on the gender configuration of the doctor and the patient.

DIMENSION OF SOCIAL CONVICTIONS

To determine whether, in the opinion of the public - (10) adolescents and (11) parents of adolescents - preventive genital examination of adolescents is permitted by law, and to determine whether, in the opinion of the respondents, this right depends on the gender of the patient, the gender of the doctor, and their particular configurations.

DIMENSION OF GENDER PREFERENCE

To determine whether adolescents have a gender preference for physicians performing the following procedures on them (12) chest auscultation, (13) weight measurement, (14) posture examination, (15) genital examination.

And to determine, whether these preferences are influenced by health status, e.g., for (16) genital examination, features of infection.Questionnaire development

The adolescent-directed questionnaire is a modification and development of the author's questionnaire we used in a small study in 2020 (publication 2021) on the implementation of standards of prophylactic care for adolescents.

The work on the questionnaire included a search of the Polish paediatric standards in order to develop a list of essential elements of a preventive visit, mainly the physical examination and topics that the doctor should, according to the standards, raise during the medical interview. Polish and international recommendations for specific adolescent-friendly health care were also analysed. Two focus-like meetings with adolescents (two groups of high school students with approximately 30 participants each) were organised, during which - by asking non-suggestive open questions - a list of elements of a preventive visit that are most desired by adolescents and those that contribute to a negative evaluation of such visits was obtained. Most of the negative experiences were linked, either to the lack of conditions for a confidential conversation with the doctor or the inappropriate conduct of the examination of the genital area. Relevant questions were included in the survey.

The draft version of the questionnaire was analysed in depth and modified by two paediatric specialists. Subsequently, the questionnaire was pilot tested on a small group of adolescents from high schools in Warsaw and the surrounding area who had attended a preventive medical visit 12-18 months previously. Approximately 45 responses were obtained. In a survey conducted using the same questionnaire 2 weeks later on the same group of respondents (re-test method), responses were obtained from approximately 35 participants. In terms of questions about facts (e.g. demographics or the fact that a specific physical examination had been performed), the repetition of responses was obtained at a very high level - typically 90-95%; in terms of questions about topics raised by the doctor during the medical interview - about 90% and only slightly lower repetition in terms of questions about subjective evaluation of the visit (e.g. evaluation of the doctor's involvement). At this step, a few questions with the lowest repetition rate were eliminated from the questionnaire, e.g. the question about whether the doctor performed a palpation of the thyroid gland (repetition rate of about 70%) or the question about whether the doctor asked about the patient's general well-being.

The analysis of the answers given by the participants in the pilot test showed statistically significant relationships between the answers regarding the examination of the genital areas and their impact on the subjective evaluation of the visit, motivation to see the doctor again and declared emotions.

The questionnaire was considered to be internally consistent and reliable.

***

Translations of the questionnaires addressed to adolescents and their parents are attached below. It should be taken into account that this is not a ready-to-use questionnaire validated for use in English.

## The questionnaire for adolescents

| no | questions | possible answers |
| --- | --- | --- |
| Hi  We are researchers from the Department of Social Medicine and Public Health at the Medical University of Warsaw. We are currently investigating the relationship between adolescents and primary care physicians. We would like to know whether this care is delivered according to standards and whether doctors respect the subjectivity of young patients. We would like to ask you to participate in a survey on preventive visits.  If you do not know what a preventive visit is or are interested in further information about our survey, please visit our website at www… | | |
| 1 | We now ask you to read the terms and conditions of participation in our survey and consider whether you want to take part.  The basic principles are:  • Your participation in the survey is completely voluntary. If you do not complete the survey, you will not face any negative consequences.  • You remain completely anonymous - the survey does not ask for any details that identify you, e.g. your name, school or town of residence.  • Even if you decide to take part in the survey, you can discontinue at any time. We will still be grateful that you made the attempt.  • No one will know whether you complete or stop the survey - neither parents nor representatives of your school.  • We would very much like to ask you to answer honestly.  • The questionnaire, as well as the entire study, has been approved by the Bioethics Committee of the Medical University of Warsaw, and a team including pediatricians worked on its preparation. We are confident that completing the questionnaire will not expose you to excessive stress or any other unpleasantness. However, if you feel that we have asked you about difficult or unpleasant matters that have caused you negative emotions, please contact us and we will help you.  • You can also contact us for other reasons relating to the study. Call or write (contact details).  For more information (link to website here). | I voluntarily agree to participate in the study and to complete the survey  I do not agree to participate in the survey |
| 2 | - I am ... | a teenager and I have been to the doctor for a preventive check-up within the last 18 months  a teenager and I have NOT been to a doctor for a preventive check-up in the last 18 months |
| To start, a series of quick questions about your last preventive care visit to your doctor. First, we will ask you about any topics you may have raised with your doctor during the conversation. Then we will ask a series of questions about the physical examination you had. | | |
| 3 | Have you talked with the doctor, about …  your performance at school, e.g. asking about grades or any difficulties? | Yes  No  I can’t remember |
| 4 | the time you spend in front of a screen, e.g. a mobile phone or computer? | Yes  No  I can’t remember |
| 5 | alcohol and the consequences of drinking it? | Yes  No  I can’t remember |
| 6 | smoking and the consequences of smoking cigarettes? | Yes  No  I can’t remember |
| 7 | sexual activity for example, asking you about your experiences in this area, your plans for the near future or any concerns you would like to address? | Yes  No  I can’t remember |
| 8 | drugs or recreational use of medicines that affect the mental ability - the consequences of their abuse or ways to reduce the problem? | Yes  No  I can’t remember |
| 9 | your mood, possible symptoms of chronic sadness, lack of motivation, symptoms of possible depression or suicidal thoughts? Maybe he/she gave you a special text/questionnaire to fill in on the subject? | Yes  No  I can’t remember |
| 10 | violence (physical, psychological, sexual) or feeling threatened by such violence? | Yes  No  I can’t remember |
| 11 | the use of electronic cigarettes and the consequences of using them? | Yes  No  I can’t remember |
| 12 | the length and quality of your sleep and the factors that affect it? | Yes  No  I can’t remember |
| 13 | healthy eating? | Yes  No  I can’t remember |
| 14 | your physical activity, e.g. the sports you do - frequency and length of training, intensity, type? | Yes  No  I can’t remember |
| 15 | contraception, e.g. the pros and cons of different methods? Or has your doctor explicitly recommended a method to you? | Yes  No  I can’t remember |
| 16 | your relationships with peers, e.g. do you have friends in or out of school? | Yes  No  I can’t remember |
| 17 | flu vaccinations and/or other recommended (non-compulsory) vaccinations? | Yes  No  I can’t remember |
| 18 | reproductive system complaints, even potential ones but requiring an urgent visit to hospital? | Yes  No  I can’t remember |
| 19 | menstruation or ejaculations? | Yes  No  I can’t remember |
| 20 | addictions of your family members, e.g. to alcohol or cigarettes? | Yes  No  I can’t remember |
| 21 | your gender identity and sexual orientation? | Yes  No  I can’t remember |
| 22 | Has your doctor encouraged you to have sexual abstinence, e.g. outlined its benefits? | Yes  No  I can’t remember |
| 23 | During your last doctor's visit, did he or she suggest that your parent leave the office to talk ‘one-on-one’ without witnesses and more frankly? | Yes  No  I can’t remember |
| During a preventive visit, the doctor may carry out various physical examinations. Not everyone is examined in the same way. What was it like for you? | | |
| 24 | Did the doctor auscultate your chest with a stethoscope? | Yes  No  I can’t remember |
| 25 | Did the doctor examine your abdomen (by pressing your abdomen while you were lying down)? | Yes  No  I can’t remember |
| 26 | Did the doctor examine your posture (spine) - watching your back, asking you to bend over? | Yes  No  I can’t remember |
| 27 | Before examining your posture (spine), did the doctor ask you if he or she could carry out the examination in the presence of a parent? | Yes  No  I can’t remember  no such examination was carried out at all) |
| 28 | Did the doctor assess your degree of puberty, i.e. in the case of boys he looked at the testicles and penis and pubic hair, and in the case of girls at the pubic hair and breasts (without underwear)? | Yes  No  I can’t remember |
| 29 | Did the doctor examine your genitals, i.e. in the case of boys, palpate each of the testes and retract the foreskin (or ask you to retract it), in the case of girls, spread your labia with your fingers? | Yes  No  I can’t remember |
| 30 | Before examining your genitals or degree of maturity, did the doctor ask your consent for this particular examination and explain its purpose? (‘yes’, ‘no’, ‘I don't remember’, ‘such an examination was not carried out at all’) | Yes  No  I can’t remember  no such examination was carried out at all’) |
| 31 | Were you physically separated from your caregiver/parent during the genital/maturity examination, e.g. by a screen or was your parent asked to leave? | the doctor put up a screen or asked the parent to leave the office  the parent looked away but we were not physically separated by anything  the parent saw the whole examination  I don't know / I don't remember / there was no such examination) |
| 32 | Your intimate areas were exposed .... | only a short moment necessary for the genital examination  longer, the doctor then also performed other examinations, e.g. a spinal examination  I do not remember  there was no such examination during which my intimate zones were exposed |
| Now it is time for a more synthetic evaluation of your last preventive visit | | |
| 33 | Evaluate how the doctor respected your intimacy during your last balance visit | 5-points Likert scale |
| 34 | Rate the physician's interest in what you had to say to him or her during your last preventive visit | 5-points Likert scale |
| 35 | Rate the benefit you got from your last preventive visit | 5-points Likert scale |
| 36 | What is your overall assessment of your last preventive medical visit | 5-points Likert scale |
| 37 | How did your last visit affect your motivation to attend your next preventive visit? | 5-points Likert scale |
| 38 | Of the following emotions, please select the ones you associate with your last preventive visit to the doctor (please indicate no fewer than two). | calmness, joy, acceptance, fear, trust, surprise, thoughtfulness, sadness, boredom, disgust, anger, annoyance, anticipation, curiosity  (emotions displayed in random order) |
| 39 | Who accompanied you to the office? | (Mum/Dad/I was alone/ I don't remember) |
| 40 | At your last preventive visit, you were with: | a male physician  a female physician  I don't remember the gender of the physician |
| 41 | What is your relationship with the mentioned physician? | he/she is a member of my family or a friend of my parents  he/she is my ‘permanent’ physician  he/she is NOT my ‘permanent’ physician |
| 42 | Does this practitioner also care for adult members of your family? | (yes/ no/ don't know) |
| 43 | How long did your last visit last? | (< 10 minutes / 10-14 minutes / 15-19 minutes / 20-24 minutes / 25 minutes or more) |
| 44 | How likely would you be to recommend this doctor to a good friend of yours? | (scale from 0 to 10) |
| A preventive medical visit is not only a physical (body) examination, but also a conversation during which the doctor should find out about and prevent circumstances that threaten the health of adolescents, e.g. by giving advice or answering questions.  Imagine that tomorrow you go to a preventive appointment with your doctor. The physician has suggested a list of topics. Decide which of the topics concerns you directly to some extent (e.g. you smoke cigarettes and would like medical advice on how to combat your addiction) or could realistically concern you in the near future (e.g. you are considering becoming sexually active and would like medical advice on which contraceptive method to use).  Using a five-point scale (I don't need to talk about this topic, I rather don't need to talk about this topic, I don't have an opinion, I rather need to talk about this topic, I need to talk about this topic) refer to the suggestions for each topic.  (topics displayed in random order) | | |
| 45 | your performance at school | 5-points Likert scale |
| 46 | the time you spend in front of a screen, e.g. a mobile phone or computer | 5-points Likert scale |
| 47 | alcohol consumption and the consequences of drinking it | 5-points Likert scale |
| 48 | smoking and the consequences of smoking cigarettes | 5-points Likert scale |
| 49 | sexual activity | 5-points Likert scale |
| 50 | drugs or recreational use of drugs that affect the mental ability | 5-points Likert scale |
| 51 | your mood, possible symptoms of chronic sadness, lack of motivation, symptoms of possible depression or suicidal thoughts | 5-points Likert scale |
| 52 | violence (physical, psychological, sexual) or feeling threatened by such violence | 5-points Likert scale |
| 53 | the use of electronic cigarettes and the consequences of using them | 5-points Likert scale |
| 54 | the length and quality of your sleep and the factors that affect it | 5-points Likert scale |
| 55 | healthy eating | 5-points Likert scale |
| 56 | your physical activity, e.g. the sports you do - frequency and length of training, intensity | 5-points Likert scale |
| 57 | contraception, e.g. the pros and cons of different methods | 5-points Likert scale |
| 58 | your relationships with peers | 5-points Likert scale |
| 59 | flu vaccinations and/or other recommended (non-compulsory) vaccinations | 5-points Likert scale |
| 60 | reproductive system complaints | 5-points Likert scale |
| 60 | menstruation or ejaculations | 5-points Likert scale |
| 60 | the possible addictions of your family members, e.g. to alcohol or cigarettes | 5-points Likert scale |
| 63 | your gender identity and sexual orientation | 5-points Likert scale |
| 64 | sexual abstinence, e.g. its benefits | 5-points Likert scale |
| 65 | To the best of your knowledge, is it compulsory for teenagers to have a preventive visit to the doctor? | Yes, under current Polish law it is obligatory.  It is not compulsory, but without such an assessment the municipality may withhold the payment of ‘500+’ (a benefit of PLN 500 paid from the public budget every month to the parents of all children)  No, it is not obligatory |
| 66 | Does a male doctor have the right to examine the genitals of a teenage girl during a preventive visit? | the doctor has the right to do so  the law prohibits it |
| 67 | Does a male doctor have the right to examine the genitals of a teenage boy during a preventive visit? | the doctor has the right to do so  the law prohibits it |
| 68 | Does a female doctor have the right to examine the genitals of a teenage girl during a preventive visit? | the doctor has the right to do so  the law prohibits it |
| 69 | Does a female doctor have the right to examine the genitals of a teenage boy during a preventive visit? | the doctor has the right to do so  the law prohibits it |
| Doctors can easily distinguish between boys and girls for whom even complete undressing for a medical examination is not a problem and those for whom mere examination by a doctor of the opposite sex is uncomfortable. Neither of these attitudes is better or worse.  Very often boys and girls try to hide their embarrassment. Embarrassment is a recursive emotion - that is, people who are embarrassed are ashamed of the fact that they are embarrassed and will not admit their emotions. Nevertheless, we ask you very much to respond honestly. | | |
| 70 | By moving the slider to the right or left, select the doctor's preferred gender for a particular examination. The more you care about a particular choice, the further you move the slider.  (individual examinations displayed to respondents in random order)  o Stethoscope auscultation of the heart  o Weight measurement  o Posture (spine) examination  o Posture examination, but imagine that you are significantly overweight  o Genital examination.  o Examination of the genitals, but imagine you have an infection, there is itching and a discharge with a unpleasant smell | Scale from -5 to +5, where a value of -5 was assigned the phrase ‘I am very concerned about being examined by a doctor of the gender that is the same as my gender’, a value of +5 was assigned the statement ‘I am very concerned about being examined by a doctor of the gender that is opposite to my gender’, and a value of 0 was assigned the phrase ‘I have no preference in this matter’ |
| 71 | Here is a list of several medical procedures, please arrange them in order from those you are most willing to undergo to those that evoke the most negative emotions and associations in you.  (the procedures were displayed in a random order and the respondent was able to arrange them in the order of his/her choice)  o Eye drops  o Vaccination (injection into the shoulder muscle)  o Collection of blood for examination by a nurse of your gender  o Collection of blood for examination by a nurse of the opposite gender  o Tooth drilling at the dentist  o Genital examination by a doctor of your gender  o Examination of genital organs by a doctor of the opposite gender |  |
| 72 | In some countries, during a preventive visit, the parent is asked to leave the office so that the doctor and the adolescent patient can have a ‘one-to-one’ conversation for a few minutes.  What about you and your parent, would you agree to such an offer if the examination was, for example, tomorrow? | Me - yes, parent - yes  Me - yes, parent - no  Me - no, parent - yes  Me - no, parent - no |
| 73 | In some countries, the doctor is obliged to keep confidential the information he or she obtains from the teenager. He or she may inform a parent only of circumstances that represent a serious risk to the child's health and life. And you and your parent, would you agree to such a proposed rule? | Me - yes, parent - yes  Me - yes, parent - no  Me - no, parent - yes  Me - no, parent - no |
| 74 | From what age do you think an adolescent should have the right to talk to a doctor ‘face-to-face’ and keep confidentiality (under the conditions mentioned above). | from the age of 12 and even before  from the age of 13  14 years  15 years  16 years  17 years  18 and over, from the age of majority |
| At the very end, a few questions about who you are | | |
| 69 | I am… | A girl  A boy  I do not identify with any of the two genders mentioned above  I don't want to answer that question |
| 70 | I am | 13 years old  14 years old  15 years old  16 years old  17 years old |
| 71 | What type of school do you attend? | primary school  vocational school  technical school  general secondary school |
| 72 | Where do you live? | In the provincial capital  in a district capital  in another city  in a rural area |
| 73 | Select the province in which you live | list of 16 polish provinces (voivodships) to choose from |

## The questionnaire for parents

| no | questions | possible answers |
| --- | --- | --- |
| 1 | I am | mother / female legal guardian  father / male legal guardian |
| 2 | Your child we are talking about in this survey is | A girl  A boy  represents another gender or does not identify with any of the above-mentioned genders |
| 3 | How old is your child we are talking about in this study | 13 years old  14 years old  15 years old  16 years old  17 years old |
| 4 | What type of school does this child attend? | primary school  vocational school  technical school  general secondary school |
| 5 | Where does this child live? | In the provincial capital  in a district capital  in another city  in a rural area |
| 6 | Point out the province (voivodship) in which this child lives ? | list of 16 polish provinces (voivodships) to choose from |
| 7 | Does your child have a ‘permanent’ doctor as part of his/her primary care? | no, we go to random (available) doctors  yes, we have been going to a ‘ permanent’ doctor for more than 3 years  yes, we have been going to a ‘’permanent‘’ doctor for less than 3 years  difficult to say |
| 8 | My child's “permanent” doctor is… | a paediatrician specialist  general practitioner  we do not have a “permanent” doctor |
| 9 | How likely would you be to recommend this doctor to a good friend of yours? (your child's “permanent” doctor or the last one you went to with your teenager for a preventive visit) | (scale from 0 to 10) |
|  | Now some questions about your opinions and preferences | |
| 10 | Is it compulsory for teenagers to take part in preventive visits according to Polish law? | Yes, under current Polish law it is obligatory.  It is not compulsory, but without such an assessment the municipality may withhold the payment of ‘500+’ (a benefit of PLN 500 paid from the public budget every month to the parents of all children)  No, it is not obligatory |
| A preventive visit is not only a physical examination, but also a conversation (interview) during which the physician should find out about and prevent circumstances that put your teenager's health at risk.  Imagine that tomorrow your child goes for a preventive visit. The doctor has suggested a list of topics. Tick whether you would like the topic to be discussed by the doctor with your teenage child.  A 5-point Likert scale, where a value of -2 corresponds to the response ‘I definitely do not wish the doctor to discuss this topic with my teenage child’. , 0 - ‘I have no preference in this area’, +2 - ‘I strongly wish the physician would discuss this topic with my child’.  (random order) | | |
| 11 | performance at school | 5-points Likert scale |
| 12 | alcohol consumption and the consequences of drinking it | 5-points Likert scale |
| 13 | the time spend in front of a screen, e.g. a mobile phone or computer | 5-points Likert scale |
| 14 | smoking and the consequences of smoking cigarettes | 5-points Likert scale |
| 15 | sexual activity | 5-points Likert scale |
| 16 | drugs or recreational use of drugs that affect the mental ability | 5-points Likert scale |
| 17 | mood, possible symptoms of chronic sadness, lack of motivation, symptoms of possible depression or suicidal thoughts | 5-points Likert scale |
| 18 | violence (physical, psychological, sexual) or feeling threatened by such violence | 5-points Likert scale |
| 19 | the use of electronic cigarettes and the consequences of using them | 5-points Likert scale |
| 20 | the length and quality of sleep and the factors that affect it | 5-points Likert scale |
| 21 | healthy eating | 5-points Likert scale |
| 22 | physical activity, e.g. the sports you do - frequency and length of training, intensity | 5-points Likert scale |
| 23 | contraception, e.g. the pros and cons of different methods | 5-points Likert scale |
| 24 | relationships with peers | 5-points Likert scale |
| 25 | flu vaccinations and/or other recommended (non-compulsory) vaccinations | 5-points Likert scale |
| 26 | reproductive system complaints | 5-points Likert scale |
| 27 | menstruation or ejaculations | 5-points Likert scale |
| 28 | the possible addictions of family members, e.g. to alcohol or cigarettes | 5-points Likert scale |
| 29 | gender identity and sexual orientation | 5-points Likert scale |
| 30 | sexual abstinence, e.g. its benefits | 5-points Likert scale |
| 31 | Which of the following emotions do you think your child feels about a preventive visit to the doctor. (choose no less than two) Of course, do not ask your child - answer based on your own knowledge/intuition. | calmness, joy, acceptance, fear, trust, surprise, thoughtfulness, sadness, boredom, disgust, anger, annoyance, anticipation, curiosity  (emotions displayed in random order) |
| Which physician - according to Polish law - can examine the genital organs of a teenager as part of a preventive examination, even if he/she does not report any worrying symptoms? (random order) | | |
| 32 | a male doctor - a teenage girl | the doctor has the right to do so  the law prohibits it |
| 33 | a male doctor - a teenage boy | the doctor has the right to do so  the law prohibits it |
| 34 | a female doctor - a teenage girl | the doctor has the right to do so  the law prohibits it |
| 35 | a female doctor - a teenage boy | the doctor has the right to do so  the law prohibits it |
| 36 | In some countries, during a preventive visit, the parent is asked to leave the office so that the doctor and the adolescent patient can have a ‘one-to-one’ conversation for a few minutes.  What about you and your teenager, would you agree to such an offer if the examination was, for example, tomorrow? | Me - yes, teenager - yes  Me - yes, teenager - no  Me - no, teenager - yes  Me - no, teenager – no  (random order) |
| 37 | In some countries, the doctor is obliged to keep confidential the information he or she obtains from the teenager. He or she may inform a parent only of circumstances that represent a serious risk to the child's health and life. And you and your teenager, would you agree to such a proposed rule? | Me - yes, teenager - yes  Me - yes, teenager - no  Me - no, teenager - yes  Me - no, teenager – no  (random order) |
| 38 | From what age do you think an adolescent should have the right to talk to a doctor ‘face-to-face’ and keep confidentiality (under the conditions mentioned above). | from the age of 12 and even before  from the age of 13  14 years  15 years  16 years  17 years  18 and over, from the age of majority |

Both the questionnaires addressed to the adolescents and their parents contained 4 questions each allowing the generation of unique codes to pair the questionnaires completed by a particular adolescent with the answers given by his/her own parent. These related to: the season of the teenager's birth, the month of his/her mother's birth, the first letter of the grandmother's name on the mother's side, the last digit of the teenager's telephone number). Answering these questions was not compulsory. Despite obtaining responses from 1,072 adolescents and from 685 parents, the aforementioned 4 questions remained unanswered in the vast majority of cases. As a consequence, only 17 pairs of matching codes, corresponding to parent-teenage pairs, could be identified. Due to the low number of such pairs, the analysis of the concordance of responses in the parent-teenager system was omitted.

Both questionnaires included questions on topics not discussed in this publication - i.e. adolescents' emotions, respect for the right to confidentiality and topics of conversation with the doctor. The data obtained through these questions are being analysed and may be published later. In the questionnaire, these questions have been included not only to obtain specific data, but also to ‘dilute’ the questions on sex, gender, examination of genital regions, physical nakedness and respect for the right to intimacy. In our experience, an excessive focus on questions about sex and nakedness (even during a medical examination) would be perceived as unacceptable in traditional Polish society, and thus would reduce the chance of obtaining consent from schools and parents of adolescents for their participation in the survey.

### Quotation from Polish standards on examination of genital areas during a preventive visit:

Girls

(the doctor is obliged) "To examine the external genital organs during all preventive examinations. Paying attention to possible abnormalities (e.g. redness and swelling of the vulva, vaginal discharge, malformations, bruising, epidermal lesions in the genital area and inner surface of the thighs, and other symptoms that may indicate sexual abuse)."

boys

**Preparation for the test.** Explain to the boy why and how you will carry out the examination. Ensure privacy and ask him to undress completely. Do the test standing up with warm hands.

**Examination of penis.** Pull back the foreskin (at the end of puberty it should be completely retractable). Note whether there are any malformations or ulcers. Using gentle pressure with the index finger and thumb, open the orifice of the urethra. Check for redness of the mucous membranes, ulcers, scars and condylomas.

**Examination of the scrotum and testes.** Observe and palpate the entire skin surface of the scrotum (by pulling the scrotum upwards, the posterior surface of the scrotum may be inspected). Gently palpate the testes, epididymis and spermatic cords with the index finger and thumb to check for palpable lesions and pressure soreness.

### Adolescents’ preferences regarding the gender of the doctor

Distribution of preferences regarding the gender of the doctor conducting **“non-intimate” examinations**.


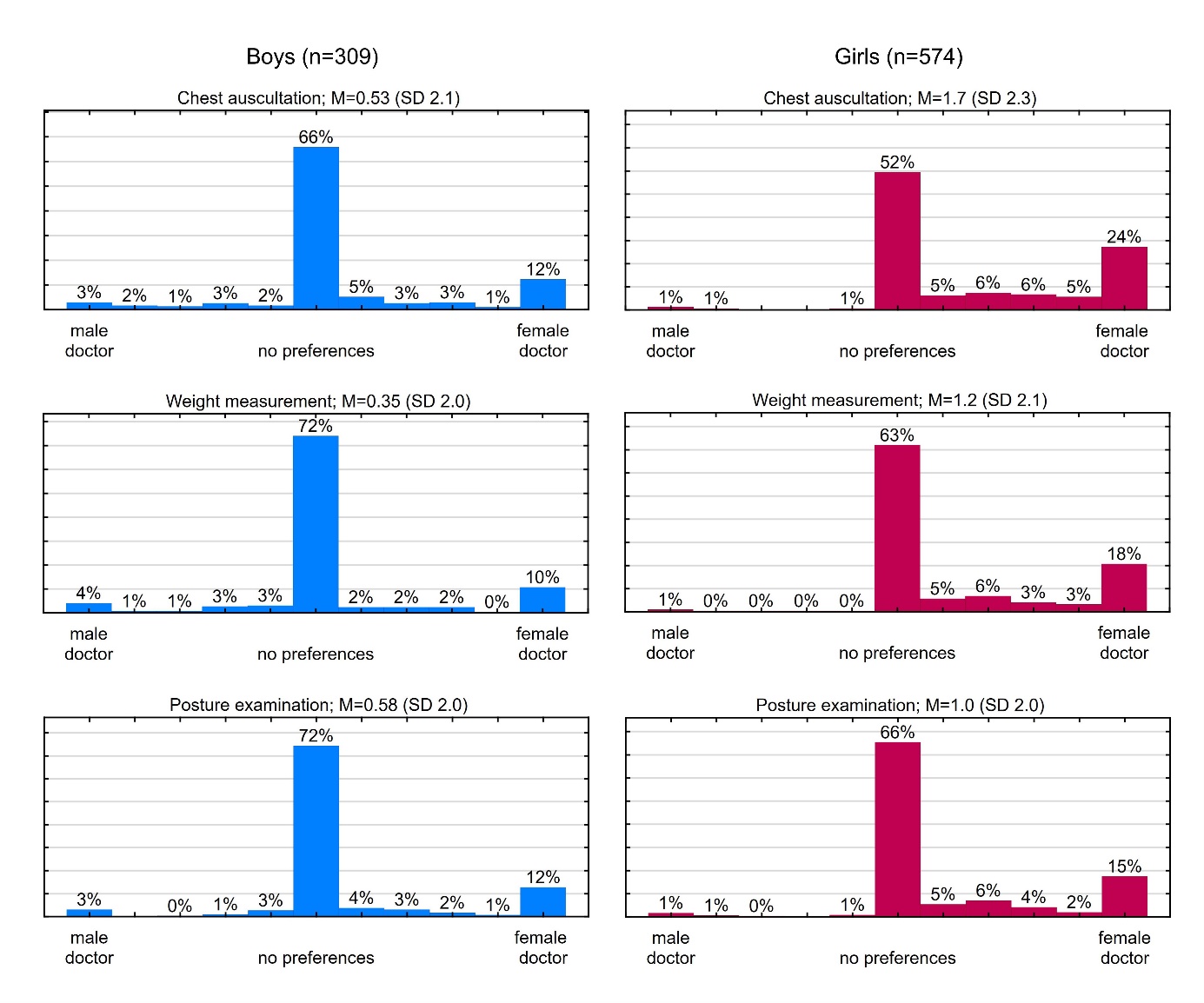


The respondents indicated the preferred gender and the strength of this preference on a scale from -5 to 5 (left side of Zero when they preferred a male doctor and right side when they preferred a female doctor).
